# A Brief Review of Transparent Conducting Oxides (TCO): The Influence of Different Deposition Techniques on the Efficiency of Solar Cells

**DOI:** 10.3390/nano13071226

**Published:** 2023-03-30

**Authors:** Ganesh T. Chavan, Youngkuk Kim, Muhammad Quddamah Khokhar, Shahzada Qamar Hussain, Eun-Chel Cho, Junsin Yi, Zubair Ahmad, Pitcheri Rosaiah, Chan-Wook Jeon

**Affiliations:** 1 School of Chemical Engineering, Yeungnam University, Gyeongsan 38541, Gyeongbuk, Republic of Korea; gtchavan1992@gmail.com; 2 College of Information and Communication Engineering, Sungkyunkwan University, Suwon 16419, Gyeonggi-Do, Republic of Korea; 3 Department of Electrical and Computer Engineering, Sungkyunkwan University, Suwon 16419, Gyeonggi-Do, Republic of Korea; 4 Department of Physics, COMSATS University Islamabad, Lahore Campus, Lahore 54000, Pakistan; 5 Applied College, Mahala Campus, King Khalid University, P.O. Box 9004, Abha 61413, Saudi Arabia; 6 Unit of Bee Research and Honey Production, Faculty of Science, King Khalid University, P.O. Box 9004, Abha 61413, Saudi Arabia; 7 Department of Physics, Saveetha School of Engineering, Saveetha Institute of Medical and Technical Sciences (SIMATS), Thandalam, Chennai 602105, India

**Keywords:** TCOs, ITO thin films, deposition techniques, optical properties, silicon heterojunction solar cells

## Abstract

Global-warming-induced climate changes and socioeconomic issues increasingly stimulate reviews of renewable energy. Among energy-generation devices, solar cells are often considered as renewable sources of energy. Lately, transparent conducting oxides (TCOs) are playing a significant role as back/front contact electrodes in silicon heterojunction solar cells (SHJ SCs). In particular, the optimized Sn-doped In_2_O_3_ (ITO) has served as a capable TCO material to improve the efficiency of SHJ SCs, due to excellent physicochemical properties such as high transmittance, electrical conductivity, mobility, bandgap, and a low refractive index. The doped-ITO thin films had promising characteristics and helped in promoting the efficiency of SHJ SCs. Further, SHJ technology, together with an interdigitated back contact structure, achieved an outstanding efficiency of 26.7%. The present article discusses the deposition of TCO films by various techniques, parameters affecting TCO properties, characteristics of doped and undoped TCO materials, and their influence on SHJ SC efficiency, based on a review of ongoing research and development activities.

## 1. Introduction

In the last few years, every continent has been affected by weather anomalies, such as record-high or record-low temperatures, an increased rate of hurricanes and typhoons, drought, and flooding [1,2]. Therefore, it is critical and significant for mankind to take serious steps to reduce its carbon footprint [1,2,3]. This can be done by using renewable resources that meet our energy needs with minimal carbon footprints or carbon-free outcomes. Some of the popular renewable energy resources are hydropower, biofuels, solar energy, wind power, biomass, and geothermal energy [1,2,3]. Of these, solar energy is the most reliable, cheapest, and simple to use [1]. In contrast to fossil fuel power-generating procedures, solar power generation causes negligible greenhouse gas emissions and other pollutants during its operations. 

It is assumed that, from 2012–2040, the world’s energy needs will be enhanced by 48% and released carbon dioxide will increase by 34% [1]. To date, a variety of solar cells, including crystalline silicon (c-Si), dye-synthesized SCs (DSSCs), amorphous silicon (a-Si), hybrid SCs, monocrystalline SCs (mono-Si), polycrystalline SCs, nanocrystalline SCs, multi-junction SCs, perovskite SCs, organic SCs, plasmonic SCs, photoelectrochemical cells (PECs), quantum dot SCs (QDSCs), solid-state SCs, and graded bandgap multilayer SCs have been thoroughly studied [4,5,6,7]. Organic SCs are gaining popularity due to their intrinsic properties, such as their light weight, their mechanical flexibility, and their transparency, as well as their low manufacturing costs [5,6,7]. Recently, a c-Si-based SC showed a high efficiency of 25.6%, which was realized by silicon heterojunction (SHJ) technology [8]. However, the cost of manufacturing the c-Si SC was high. Therefore, the realization of high efficiency in c-Si SCs at a reasonable cost requires further attention. An efficient method is to deposit transparent conducting oxide (TCO) film layers on the c-Si substrates [9].

In a previous study, TCO films were engaged to establish contact between doped a-Si:H films and metallic elements to complete the cell arrangement [10]. Generally, a TCO plays two roles in solar cells: (1) TCO film collects the charge carriers and acts as an electrode, and (2) the front TCO film also acts as an anti-reflection coating [11]. It was found that the refractive index, mobility, and extinction coefficient of TCO layers govern the short circuit current (J_sc)_ and fill factor (FF) in SCs [12]. In addition, highly conductive TCO electrodes play a key role in decreasing the parasitic absorption losses in SCs [13]. Thus, the fabrication of efficient and economical Si-based thin-film solar cells requires a TCO layer with excellent optoelectrical properties [11].

Recently, research and development activities have been concentrated on back and front contact electrodes to enhance the efficiency of SHJ solar cells. Notably, undoped zinc oxide (ZnO) [14,15], Ga or Al-doped ZnO (GZO or AZO) [16,17,18,19], hydrogen-doped ZnO [20], F-doped SnO_2_ (FTO) [21,22], and Sn-doped In_2_O_3_ (ITO) [23] thin films have excellent optoelectrical properties. In addition, they are suitable to fabricate a window layer of SCs [24,25]. Hydrogenated indium oxides (IO:H), tungsten-doped hydrogenated indium oxides (IWO:H), and cerium-doped hydrogenated indium oxides (ICO:H) with high mobilities (>100 cm^2^/V·s), energy band gaps between 3.5–3.8 eV, high conductivity, and combinations of broadband transparency are being used [13].

ITO is currently the best choice for TCO electrode materials because of its very high transmission (>80%), high conductivity (10^4^ Ω^−1^ cm^−1^), low light absorption, low refractive index, high stability, and toughness. It is being used in a variety of optoelectronic applications, such as electronic display, heaters in windows, architectural uses, automotive uses, sensors, flat panel displays, light bulb glass, and SCs [26,27,28]. However, a few critical issues, such as lower surface energy and thermal and chemical instability, restrict the widespread application of ITO layers [29]. Moreover, HSCs need further development in the surface and optical properties of ITO layers [30,31]. Other dopant elements, such as Cu, Ce, Ag, Zr, Fe, W, Fe, Ta, Hf, Nb, Mo, and Ti, have been doped into polycrystalline In_2_O_3_ films to decrease the electrical resistivity and increase the mobility and transmittance of TCO thin films [30,31,32,33,34,35]. The optoelectrical characteristics of ITO electrodes strongly rely on the concentrations of impurities and the stoichiometric control of the surface microstructure. 

High-temperature deposition techniques show a high conductivity and transmittance, along with the formation of defects in the p-layer from hydrogen effusion. Hence, the fabrication of ITO-based front electrodes at low deposition temperatures is identical to fabricating highly efficient PV cells [36]. In SHJ cells, a low sheet resistance was obtained by a very thin emitter layer, and the ITO front contact electrode was used to collect photogenerated currents [37]. Hussain et.al. reported that ITO electrodes are normally useful for the fabrication of a window layer in SHJ SCs [38]. Much attention was paid to optimizing the optoelectronic and anti-reflective properties of ITO by varying the deposition parameters, such as deposition temperatures, oxygen content, and layer thickness. Of the different deposition methods, sputtering is the best technique to enhance the FF and J_sc_ of SHJ [37]. Still, several TCO characteristics should be improved to realize highly efficient PV technologies [39]. For PV applications, the choice of suitable TCO electrodes is focused on band alignment, work function, materials compatibility, composition, and processing costs. The present article provides a brief review of doped ITO materials, with different strategies to advance the efficiency of SHJ SCs.

## 2. Deposition of ITO Thin Films by Various Techniques

ITO thin film layers can be grown using several fabrication methods, such as nebulizer spray pyrolysis (NSP) [40], spray pyrolysis [41], pulsed laser deposition [42], thermal evaporation [43], chemical sol-gel processes [44], magnetron sputtering [45,46], and vapor deposition [47]. Each technique has its advantages and disadvantages [48]. Table 1 reveals the deposition of ITO thin films by various techniques with its merits and demerits [14,15,36,38,40,41,42,47]. However, radio frequency (RF) and direct current (DC) magnetron sputtering are the best techniques for industrial-level device fabrication, due to their large-area deposition, high deposition rate, and good reproducibility [14,15].

The In_2_O_3_ thin layers with different Sn-doping content were deposited via the reactive thermal evaporation (RTE) method [36]. The deposition conditions were as follows: the base pressure was 29 × 10^−3^ Pa; the substrate-to-source distance was 21 cm; the O_2_ flow rate was 178 sccm; the growth rate was 0.01 nm/s; and the deposition temperature was 160 °C. The 6.0 wt% Sn-doped ITO revealed optimum performance, with high Hall mobility of 47 cm^2^·V^−1^·s^−1^, high transmittance of 87%, a low resistivity of 3.749 × 10^−4^ Ω·cm, and 10.51% efficiency for HIT SCs [36]. Lien et al. described the synthesis of ITO thin film by e-gun evaporation with the ITO pellet target having 95 wt% In_2_O_3_ and 5 wt% SnO_2,_ which later resulted in 16.4% efficiency for HJ SCs [49]. The ITO electrodes were synthesized on silicon (100) and glass substrates at the subsequent synthesis parameters: base pressure = 6.7 × 10^−4^ Pa and process pressure of 1.3 × 10^−3^ Pa in the chamber, an O_2_ flow rate of 0.6 sccm, an 0.2 nm/s growth rate, and varying substrate temperatures from 100 °C to 300 °C. After optimizing deposition conditions, the HJ solar cells fabricated using optimized ITO at 230 °C showed an efficiency of 16.4% [49]. Thirumoorthi et al. discussed the growth of the ITO layer with various Sn concentrations via nebulizer spray pyrolysis (NSP), which showed a band gap of 4.07 eV, optical transmittance of 87%, carrier concentrations of 6.1 × 10^20^ cm^−3^, and resistivity of 3.9 × 10^−4^ Ω-cm, respectively [48]. The W-doped In_2_O_3_ films were deposited using reactive plasma deposition technology and yielded a maximum cell efficiency of 22.03% for SHJ [32]. Silver-doped ITO thin layers were deposited by the sol-gel technique and showed a carrier concentration of 6.8 × 10^20^ cm^−3^, a resistivity of 2.4 × 10^−4^ Ω-cm, mobility of 37 cm^2^/V, and transmittance of 92% after annealing at 500 °C [44]. For the deposition of ITO via Zr layers was made feasible using various deposition techniques. The ITO:Zr thin layers were fabricated using a co-sputtering technique. In the deposition process, the 1 × 10^−4^ Pa vacuum and 0.5 Pa pressure were maintained. The 240 nm electrode thickness was realized after 30 min of deposition time [29]. 

Recently, our group reported the deposition of Zr:ITO film by RF magnetron sputtering. The deposited layers were engaged as a front electrode in HIT SCs, which showed high efficiency (ɳ) of 18.30% and high J_sc_ = 34.44 mA/cm^2^, V_oc_ = 710 mV, and FF = 74.8% [30]. Among the different synthesis methods of TCO (ITO) deposition, RF magnetron sputtering is the most auspicious method for SHJ SC-device applications. The primary advantages of magnetron sputtering are ease of sputtering any metal, alloy, or compound; high deposition rates, high purity, excellent coverage, uniformity, and high adhesion of films on large-area substrates; and the ability to coat heat-sensitive substrates. In general, sputtering can provide strict control on optical transmittance, large bandgaps (>3.5 eV), and high electrical conductivity by fine-tuning the deposition parameters [48,50,51]. Because of good technological maturity in its usage, film characteristics can be engineered by altering the sputtering parameters, including the O_2_ flow rate, RF power, and substrate temperature [52,53,54].

### TCO for Silicon Heterojunction Cell (Front and Rear Emitter)

**Figure 1:**
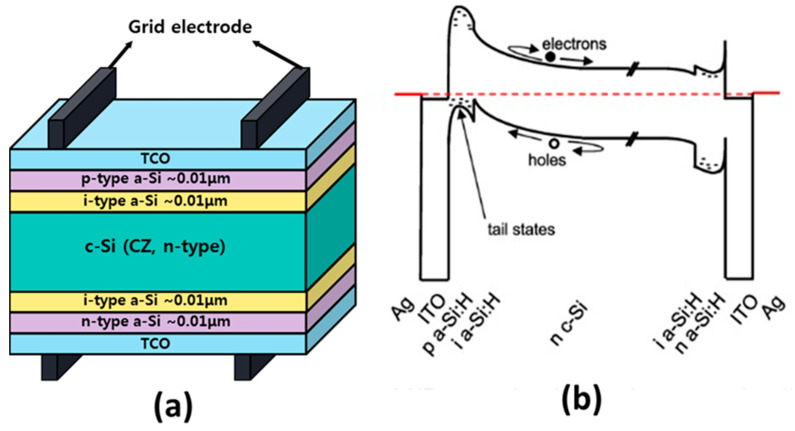
(**a**) Structure of an SHJ solar cell (reprinted with permission from ref. [61], copyright form Elsevier order number 5492900736345); (**b**) energy band diagram for an SHJ solar cell (reprinted with permission from ref. [62], copyright form AIP order number 5492901129169).

For the efficient performance of the SHJ device, the following TCO properties should be in tune with the device structure: (1) the front layer with transparency in the 350 nm to 1200 nm range [55]; (2) a refractive index ~2.0 to assist as an anti-reflection coating on top of the silicon wafer [56]; (3) deposition of the ITO, which may not prompt a variation in the a-Si/c-Si heterojunction and damage the underlying a-Si film because both cases reduce the performance [57,58]; (4) good ohmic contact with the metal electrodes [59]; (5) rear layer transparency of around 800 nm to 1200 nm to escape the absorption of the IR light [60]. Generally, SHJ silicon can absorb light with wavelengths lower than about 1100 nm (1.1 μm), which corresponds to the bandgap energy of silicon. This means that silicon can absorb a significant portion of the visible and near-infrared regions (IR) of the electromagnetic spectrum. However, for the efficient conversion of light into electrical energy in a solar cell, it is important to match the absorption spectrum of silicon with the solar spectrum. The solar spectrum has a peak intensity of around 500 nm (green light), and silicon solar cells are typically designed to absorb light in the range of 400–1100 nm, which covers most of the visible spectrum and a portion of the near-infrared (IR) spectrum. However, IR light management in silicon heterojunction solar cells is neither like that in diffused-junction crystalline silicon solar cells nor like that in thin-film silicon cells. Heterojunction cells and monocrystalline diffused-junction cells share the same random pyramid texture at the silicon surfaces so that the angular distribution of light paths—and, thus, the probability of absorption in the wafer or escape out the front—is similar in both devices in the absence of parasitic absorption. However, there is parasitic absorption in both devices, and it is different in each device. Heterojunction solar cells require a TCO layer at the front to transport charge laterally, and a TCO layer is commonly employed at the rear as well. Free carriers in these layers absorb IR light, including the 800–1200 nm photons that one would like to be absorbed in the wafer instead. Thus, in terms of IR light propagation, silicon heterojunction solar cells are like rear-passivated cells with absorbing dielectric passivation layers. Thin-film microcrystalline silicon solar cells, in contrast, have a TCO/crystalline silicon/TCO/rear reflector structure that is similar to that of heterojunction cells and, therefore, are subject to similar parasitic absorption. The TCO layer, the intrinsic layer, and the doped a-Si layer are deposited on each side of a c-Si substrate in the SHJ SC structure (Figure 1a). This type of arrangement attains a stress-free cell assembly, which is well suited to thin-substrate SCs [61]. Figure 1a shows the development of an inversion layer in the c-Si absorber following the a-Si hole contact (p-type), supplemented by a large barrier for electrons subsequent to the band bending in c-Si and the conduction band offset between c-Si and a-Si.

Figure 1b displays the energy band drawing of an SHJ SC. In this case, holes will follow the front surface of the cell and should overtake the hurdle caused by the valence band offset (VBO) among a-Si and c-Si before they can pass through the a-Si tail states into the front-doped ITO electrode and be withdrawn through the Ag fingers. Significantly, the thickness of this barrier is organized by the a-Si carrier concentration. However, defect states play a vital role in certifying hole transportation to the electrode once a p-type a-Si provides hole removal [62]. The work function of ITO, AZO, and ZnO ranges from 3.6–5.3 eV, but the feasible value is <5 eV, which is not sufficient to achieve better ohmic contact by a-Si:H (p+) and, therefore, accelerates to degradation of FF and V_OC_. Larger work function TCO materials are being examined by exploring the novel chemical composition of existing ones [63].

## 3. Impact of TCO on Cell Efficiency

In this section, we consider three perspectives of TCO layers, free electron absorption, texturing, and interstitial oxygen concentration, which impact overall solar cell efficiency. The absorption loss in NIR by the ITO layer is triggered by the free carrier absorption, which affects the overall efficiency of the cell. To overcome this carrier absorption, the carrier concentration should be very low while maintaining sufficient conductivity [64]. The surface texturing of TCO layers is the best strategy for effective light trapping, which can further improve efficiency. The rough/textured surface is useful to enhance the path length of the absorption in the cell, due to the scattering of light [65]. The scattering of light relies on several factors, including (1) the refractive index, (2) the interface roughness, (3) the wavelength of light, and (4) the light incident angle.

**Figure 2:**
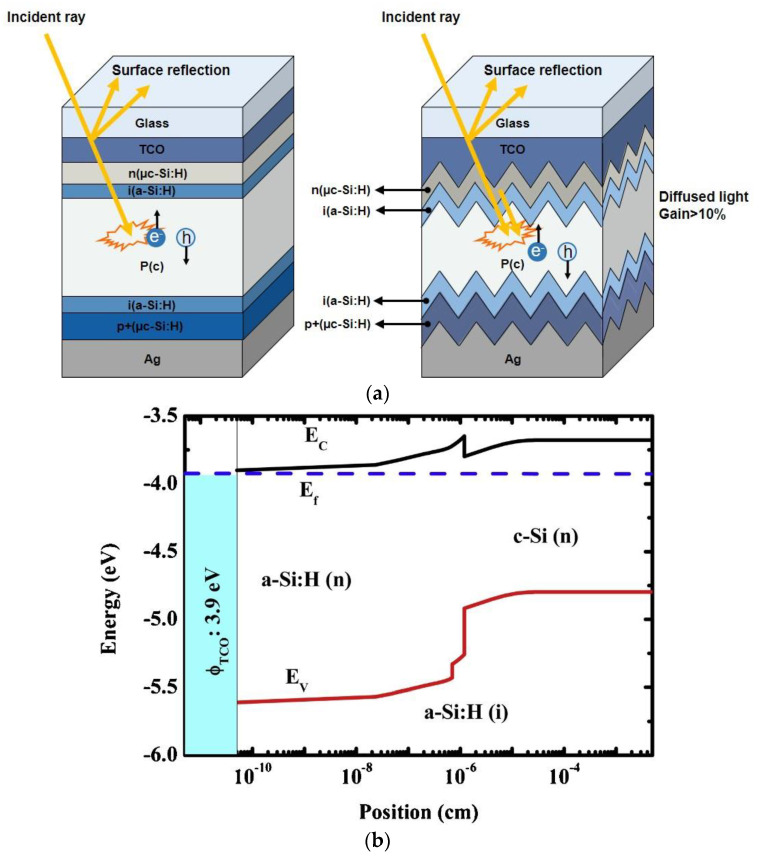
(**a**) The SHJ solar cell with flat and textured TCO layer (this figure was reprinted with permission from ref. [65]; copyright form Elsevier order number 5492910461774). (**b**) The energy band plot of diverse ITO work functions in rear-emitter SHJ SCs (this figure was reprinted with permission from ref. [66]; copyright form Elsevier order number 5492910771527).

**Figure 3:**
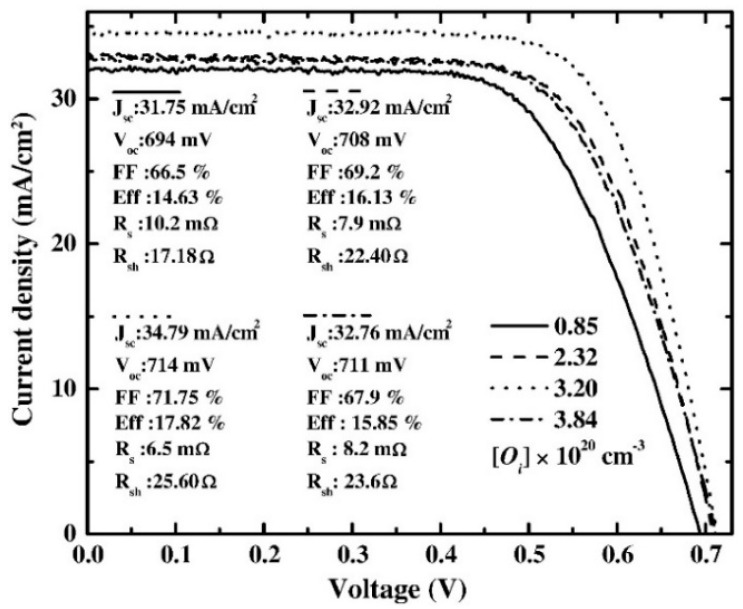
The light–CV characteristic for SHJ SCs and the influence of the diverse densities of [Oi] on the various SC parameters (reprinted with permission from ref. [67]; copyright form Elsevier order number 5492911003623).

Figure 2a displays the improved scattering of light with rough-textured surface TCO, as compared to flat TCO. Figure 2b shows that the Schottky barrier has been augmented by an improved ITO work function beneath the hypothesis of a constant electron affinity of ITO (χ: 3.9 eV) [66]. Figure 3 reveals the J_sc_ versus V_oc_ curves of the a-Si/c-Si HIT SC, which has an ITO front electrode with a dissimilar density of interstitial oxygen [Oi]. Additionally, Figure 3 shows an improvement in the cell characteristics J_sc_, V_oc_, and FF with the diverse densities of Oi. Cell efficiency is increased from 14.63% to 17.82% with an enhancement of [Oi] from 0.85 to 3.2 × 10^20^ cm^−3^. When [Oi] concentration is higher than 3.2 × 10^20^ cm^−3^, this affects the conductivity and rms roughness of ITO. Then, the leakage current is increased and, finally, cell performance is reduced [67].

### 3.1. ITO Thin Film Properties

ITO layers fabricated at 27 °C via DC and RF magnetron sputtering revealed high transparency (80–85%) and low resistance (20–25 Ω/sq at ~300 nm thickness) [68]. ITO thin film shows promising applications in different optoelectronic devices, due to some extraordinary optoelectrical properties [26,28,69]. However, ITO has the disadvantages of being expensive, providing inadequate light transmission in the blue and near UV region, being chemically unstable, displaying a weak ion barrier effect, and being mechanically brittle, which restrict its improvement. To overcome these limitations, a deliberate wrinkling technique has been used to enhance the surface properties and flexibility of ITO layers [53]. For use as transparent conductive electrodes (TCEs), TCOs are deposited on flexible substrates. As base materials of TCOs, indium oxide (In_2_O_3_), tin oxide (SnO_2_), and zinc oxide (ZnO) are typically used. By doping specific metal ions to those base materials, the transparency and conductivity noticeably increase because of the extrinsic doping effects. Among the TCOs, indium tin oxide or tin-doped indium oxide (ITO) and fluorine-doped tin oxide (FTO) are commercially available and widely used in optoelectronic devices. The deposition process of TCOs on polymer substrates such as PET and PEN is limited because of the thermal sensitivities of polymers. Flexible TCEs with higher optical transmittance and conductivity are essential for the high performance of flexible perovskite solar cells (F-PSCs). However, typically, the overall performance of plastic-based F-PSCs would be inferior to that of rigid glass-based PSCs, due to the lower optical transmittance and the lower electrical conductivity of polymer-based TCEs. The optical transmittance of substrates is very critical for power conversion efficiency (PCE) because photoactive materials should absorb as much light as possible. Polymeric materials inherently absorb ultraviolet light, and even visible light to some extent, due to the electronic transition of the chromophore in polymers, leading to light-harvesting loss for the photoactive materials. 

Zhao et al. [70] demonstrated that the flexible ITO/PET substrates show relatively lower transmittance in the visible range, compared to ITO/glass, due to the lower transparency of the polymer. The ITO is the most widely used TCO for polymer substrates, due to its better transmittance and lower sheet resistance than those of other TCOs. It is generally known that the electrical properties of an ITO strongly depend on the film composition and deposition parameters, such as sputtering power, oxygen pressure, film thickness, and substrate deposition temperature [71]. 

Zardetto et al. [72] reported that the sheet resistance of PET/ITO increases at over 180 °C, a nd the sheet resistance of PEN/ITO increases at 235 °C. However, this would not be an issue for the polymer substrates, in which a low-temperature process is essential. On the other hand, the conductivity of a plastic-based TCE employing an ITO is typically lower than that of a glass-based TCE, due to the relatively lower carrier concentration of ITO films on polymers. The microstructure of ITOs without high-temperature thermal treatments is generally amorphous or partially crystalline, leading to poor electrical properties.

### 3.2. Dopants

Kobayashi et al. described ICO:H layers as having higher μH values of 130–145 cm^2^/V s at 100-nm-thickness [34]. W-doping (3 at%) in In_2_O_3_ ceramic targets shows a low resistivity of 1.8 × 10^−4^ Ω·cm [32]. Chakraborty et al. reported that Fe-doped ITO thin films show paramagnetic behavior at room temperature [73]. Furthermore, the RF magnetron sputtering method was used to synthesize W and Ti-doped In_2_O_3_ (IWO and ITiO) electrodes at room temperature. After post-annealing, high mobility was achieved at 51 cm^2^·V^−1^·s^−1^ and 50 cm^2^·V^−1^·s^−1^ for IWO and ItiO films, respectively. However, the IWO and ItiO films showed comparatively low (80%) transmittance [74]. The optical band gap of ITO and Cu:ITO was observed at 4 eV and 3.89 eV and crystallite size 24 nm and 22 nm, respectively, but the performance of Cu:ITO thin films was not tested for SHJ solar cell application [75]. 

**Figure 4:**
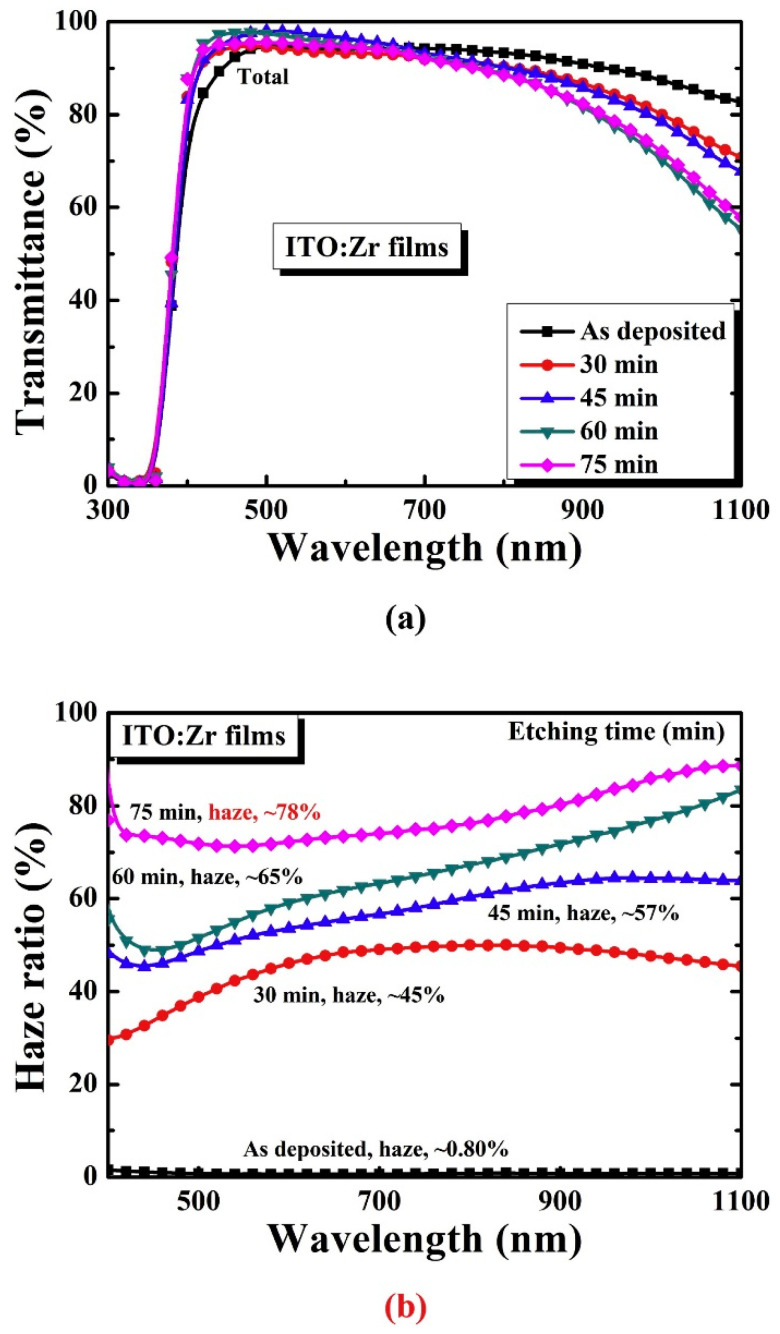
The optical characteristics (**a**) total transmittance, and (**b**) Haze ratio of ITO:Zr layers as a function of etching time. (Reprinted with permission from ref. [77]. Copyright form Elsevier order number—5492920078302).

Chen et al. achieved the growth of ITO layers with 2.5 μm thickness via metal-organic chemical vapor deposition (MOCVD) with different tin flow rates and found that MOCVD is not favorable for SHJ cell device applications [76]. Hussain et al. demonstrated an interesting analogy to decrease the overall size and enhance the performance of aSi-based solar cells using uniformly deposited TCO film that scattered more light due to a high haze ratio and high rms roughness. Figure 4a shows that once a glass-etching time is enhanced from 30 to 45 min, the transmittance of ITO:Zr films increases from 88.03% to 88.48%. Furthermore, the total transmittance of the ITO:Zr layers was reduced up to 85.55% with enhancement in glass etching time (45 to 75 min). Figure 4b reveals the haze ratio of 0.79% of as-deposited ITO:Zr in the visible wavelength region. The haze ratio of the ITO:Zr layers reached 43.35% to 73.50% when the etching time enhanced from (30 to 75 min). However, the device performance parameters of ITO:Zr showed very low J_sc_ and efficiency [77]. The graphene/ITO configuration deposited by sol-gel spin-coating reveals a boost in surface free energy from 53.826 mJm^−2^ to 97.698 mJm^−2^. Since the electrical conductivity and sheet resistance of graphene/ITO is much lower than ITO films [50].

The 4% atomic doping of Ti in ITO electrodes showed superior material properties such as low resistivity (1.6 × 10^−4^ Ω·cm), high mobility of (48.7 cm^2^·V^−1^·s^−1^), carrier concentration (8.63 × 10^20^ cm^−3^), high transmittance (92%) and a wide bandgap (3.77 eV). However, the annealing temperature of the Ti-doped ITO electrodes is 500 °C which is not suitable for SHJ solar cells [78]. The 6 wt% Sn-doped ITO electrodes showed 87% transparency, hall mobility of 47 cm^2^·V^−1^·s^−1^, and low resistivity of 3.749 × 10^−4^ Ω·cm. But unfortunately, the energy conversion efficiency of SHJ solar cells is low at around 10% [36]. Hussain et al. stated that the low O_2_ concentration improves the work function and microstructure of the Zr:ITO films while higher O_2_ concentration degrades the properties of ITO:Zr layers [31]. The Grey relational analysis shows an excellent compromise between the optoelectrical characteristics of ITO films at an operating pressure of 0.4 Pa and a power density of 0.685 W·cm^−2^. At the optimum conditions, the ITO electrodes showed 91.251% transmittance and electrical resistivity of 1.316 × 10^−4^ Ω·cm, however, the efficiency remains below 20% [79].

### 3.3. Work Function

The development of hole injection barrier from (0 to 0.129 eV) and work function from (Φ_ITO_) (4.15 to 4.30 eV) results in the progress of Voc, FF, and ɳ. The device exhibited the best performance with high FF = 0.737, V_oc_ = 635 mV, and ɳ = 14.33% for substrate temperature (Ts = 200 °C) [80]. SHJ reveals high efficiency of 17.7% with 12.7 at.% Zn content in the ITO layer and then efficiency diminished slowly once the Zn content >12.7 at.%. While the (Φ_ITO_) of TCO layers is ~5.2 eV, if Φ_ITO_ > 5.2 eV, the band flattening appears, and hence, Φ_ITO_ of the TCO layers strongly affects the performance of SHJ SCs [81]. Jian et al. carried out a simulation on TCO/n-a-Si:H/i-a-Si:H/p-c-Si/pC-a-Si:H/Ag SC using AFORS-HET software and suggested that the Φ_ITO_ of a TCO needs to be <4.4 eV to achieve a maximum efficiency (27.07%) [82]. Similarly, Wen et al. scrutinized the influence of the Φ_ITO_ of a TCO on the distribution of carrier, energy band structure, and interface recombination with the AFORS-HET program [83]. The Φ_ITO_ of the TCO strongly influenced the mobility of the carrier and the overall SC device performance [83]. The conversion efficiency textured TCO/p-type a-Si:H/i-type a-Si:H/n-type c-Si/i-type a-Si:H/n+-type a-Si:H/Al SC achieved 27.37% (JSC:41.85 mA·cm^−2^, VOC:805.5 mV, FF:81.2%) by simulation when the VBO at the n-type c-Si/BSF interface for the back surface field (BSF) was ~0.37 eV, and the VBO at the p-type a-Si:H/n-type interface was under 0.37 eV; then, yhr Φ_ITO_ for the TCO/p-type a-Si:H interface was maintained >5.2 eV with decreased interface recombination [83].

### 3.4. Sheet Resistance

**Figure 5:**
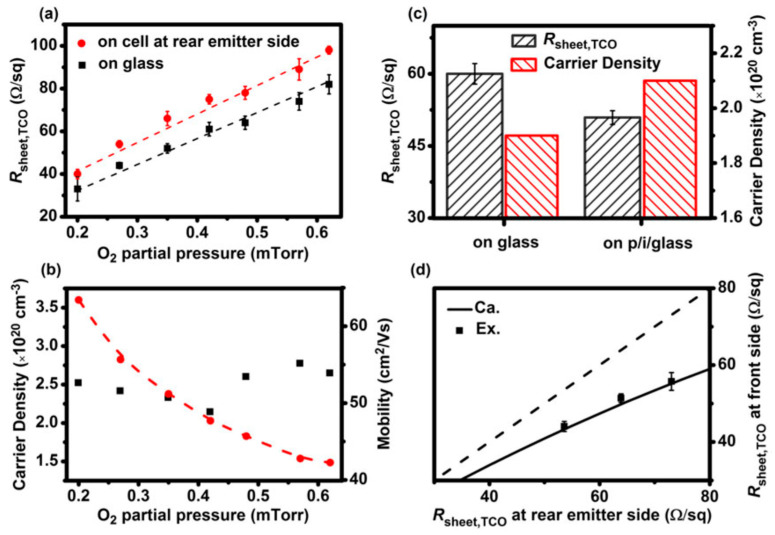
The deviations of (**a**) TCO sheet resistance measured on glass and at the rear side of the cell, (**b**) oxygen partial pressure as a function of mobility and carrier density of the TCO films. (**c**) Comparison of carrier density and TCO sheet resistance attained on glass and p/i/glass substrates after annealing treatment at 200 °C for 30 min. (**d**) Correlation between the *R*_sheet_ of the TCO measured at the front side and rear side of the cell [84]. (Reprinted with permission from ref. [84]; copyright form Wiley order number 5492920290840.)

Figure 5a shows that when the oxygen partial pressure is increased, the R_sheet_ of the TCO is also enhanced, due to the carrier density in the TCO, and the mobility of the TCO is stabilized at ~55 cm^2^/Vs (Figure 5b). The figure shows that on glass. the R_sheet_, of the TCO is less. compared to the cell. When the substrate is altered from glass to a-Si:H(p)/a-Si:H(i)/glass, the carrier density increased. From Figure 5c, it is observed that the R_sheet_ of the TCO decreased by ~15% (from 60 to 51 Ω/sq). The R_sheet_ of the TCO of the rear side of the cell is much greater than that of the front side, as shown in Figure 5d [84].

## 4. Current Challenges and Future Outlook

The improvement of TCOs has been continuing for over a hundred years, and since 1970 the ITO has come into the scenario [85,86]. The TCO (ITO) is at the heart of not only SHJ SCs but also different modern optoelectronic devices [87]. Nevertheless, the high cost, inadequate reserves, and toxicity of indium have restricted its large-scale application. Furthermore, for the large-scale fabrication of solar cells, the ITO has serious limitations and faces obstacles resulting from poor transparency and high resistivity [88]. The devel-opment of ITO performance by improving the electrical and optical characteristics is es-sential, due to the mass production of electronic devices [88,89]. Thus, many researchers focus on hunting substitutes for the ITO to achieve new transparent electrodes with high performance and earth-abundant materials [88]. Along with ITO:Zr, double layer ITO/In_2_O_3_, ZnO:B, Mo, Ti, W, and Nb-doped ITO, novel multicomponent ITOs with low sheet resistance and high transmittance should be explored in the near future.

**Figure 6:**
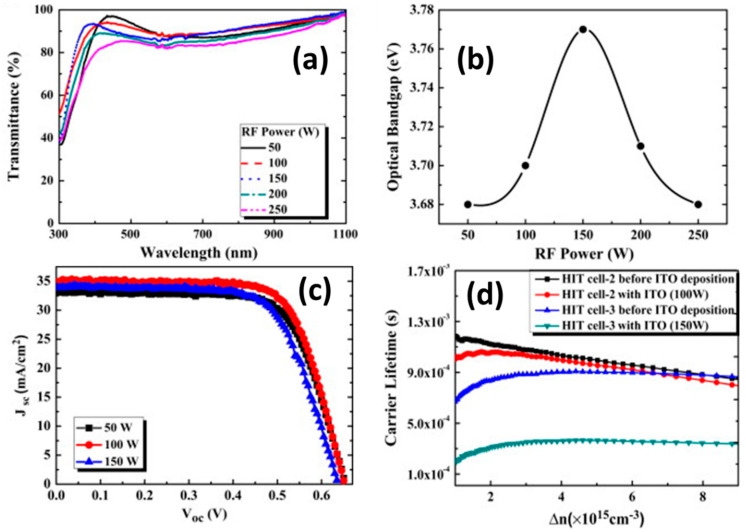
(**a**) Transmittance and (**b**) energy gap of ITO layers at different RF powers. (**c**) The J-V characteristics of SHJ PV cells for different RF powers. (**d**) Carrier lifetime for SHJ PV cell versus minority carrier density before and after deposition of ITO films for various RF powers. (Reprinted with permission from ref. [90]. Copyright form Elsevier order number 5492920513698.)

The solar cell structure contains an a-Si top junction connected with a c-Si layer by a TCO layer, and a metallic collection grid and PV modules show 19.2% efficiency. The structural and chemical stability, the low cost, and the ability to be easily textured are the precise requirements of TCOs for the construction of SHJ SCs [39]. A variation in the work function of the ITO electrodes influences the hole carrier injection flow into the ITO/a-Si:H(p) interface, which plays a crucial role in device performance [40]. Figure 6a shows the transmittance of ITO film and Figure 6b shows that the energy gap changed from 3.68 eV to 3.77 eV when the RF power varied from 50 to 250 W. The ITO electrodes were fabricated at 100 W with a high transmittance of 90.19% and low resistivity of 3.8 × 10^−4^ Ω·cm. The optimized ITO layer utilized as a front AR coating in SHJ PV cells and J-V characteristics (Figure 6c) showed a comparatively low efficiency of 16.3%.

Figure 6d shows the carrier lifetime of PV cells for different RF powers [90]. S. Ahn et al. reported that the device performance of HJ solar cells is enhanced when the [Oi] in the ITO film is increased. The highest cell parameters in this report are Jsc = 34.79 mA·cm−2, Voc = 714 mV and η = 17.82. However, the device performance is reduced when [Oi] concentration exceeds the limit from 3.2 × 10^20^ cm^−3^ [91]. One of the most important facts of the work function of TCO thin layers creates the band bending effect in TCO/a-Si:H(p) interface [92]. Zhang et al. investigated how the deposition parameters, such as the deposition temperature of the ITO, the post-annealing, and power density, affect the passivation quality of SHJ PV cells [37]. About 80 nm to 95 nm is the optimum ITO thickness value that shows high conductivity, depending on the spectral response of the device, independent of the a-Si layer thickness; hence, this analogy is useful for efficient device fabrication [52]. The ITO:Zr layers have a work function of 5.13 eV deposited at an O_2_/Ar flow ratio of 0.4%, and were used as a front AR coating in SHJ PV cells, showing improvement in the performance: Jsc = 33.66 mA·cm^−2^, Voc = 710 mV, FF = 0.724, and η = 17.31%. However, the Jsc and efficiency were still lower than those of commercial solar cells [31]. An a-Si:H/a-SiGe:H HJ solar cell developed using the RTE-deposited ITO layers showed a low efficiency of 10.51% [36]. The 82/20 nm thick ITO/In_2_O_3_-based front AR coating for SHJ solar cells demonstrated good photovoltaic behavior: Jsc = 37.42 mA·cm^−2^, Voc = 670 mV, η = 17.84%, and FF = 71.16%. The lower Voc and FF reduced cell performance [92]. 

The doping of suitable transition metal elements (Mo, Ti, Nb, and W) in In_2_O_3_ or an ITO can improve their optoelectrical characteristics significantly because of the higher mobility and smaller ionic radii [78]. The ITO:Zr/AZO layers were fabricated on plasma-textured periodic substrates utilized as a front TCO layer, and improvement in J-V characteristics was noted, but SCs still suffered from a low-efficiency problem [68,81]. Niemela et al. demonstrated >21% efficiency of large-area SHJ SCs with ALD-deposited ZnO:Al as the front- or back-side TCO [93]. Zhong et al. reported that the growth of ZnO:Al (AZO) co-sputtered with SiO_2_ to prepare AZO:SiO_2_ films with altered SiO_2_ concentration, while a c-Si SC revealed an efficiency of 19.5% with FF of 74.7% and V_OC_ of 701 mV by using AZO/Al as an electron-selective contact. These were the best outcomes among c-Si solar cells using ZnO as the electron-selective contact [94]. The 100 nm thick IO:Zr film showed a carrier density of 2.5–3 *×* 10^20^ m^−3^ electron mobility of 100 cm^2^/V·s and R_sheet_ of 25 Ω/sq. The SHJ device showed J_sc_ = 40 mA·cm^−2^, η = 23.4 efficiencies, and FF = 80% with the optimized IO:Zr front electrode [13]. The W-doped ITO (IWO) layers were fabricated via reactive plasma deposition for SHJ PV cells, having transmittance of −88.33%, carrier concentration of −2.86 × 10^20^ cm^−3^, hall mobility of −77.8 cm^2^/Vs, and resistivity of −2.80 × 10^−4^ Ω cm [32]. The W-doped indium oxide (IWO) showed a remarkable efficiency of 22.03% [32]. CeO_2_ and hydrogen co-doped In_2_O_3_ ICO:H electrodes deposited at 1% H_2_ gas flow ratio revealed a low resistivity of 2.21 × 10^−4^ Ω cm, carrier concentration of 2.01 × 10^20^ cm^−3^, and Hall mobility of 141 cm^2^/V s with a 3.84 eV bandgap. A commercial SHJ cell fabricated using ICO:H layers showed a 24.1% efficiency and 83% FF [34]. Based on the compositions, the valence states of ICO:H electrode Ce could substitute the In, which acted as a donor. The H and CeO_2_ reduced the residual strain and enhanced carrier transport. It was proved that the J_sc_, V_oc,_, and efficiency of SHJ cells may be upgraded using an ICO:H TCO electrode [34].

Wang et al. stated that wrinkling is the influential technique for achieving a high optical transmittance and a high haze ratio, which are favorable for SHJ PV cells [53]. The TCO electrodes have been broadly employed as the front electrodes for various solar cell structures, especially SHJ solar cells [95]. The Boron-doped TCO layer deposited in the low-temperatures process used in SHJ solar cells showed a stable but low efficiency of 16.6% [96]. The sputter technique was used to deposit hydrogen-doped In_2_O_3_ (IO:H) layers and post-annealing treatment was carried out below 200 °C [96]. The resulting IO:H electrodes showed some promising properties of high carrier concentration (N) = 1.5 × 10^20^ cm^−3^, mobility (μ) = 140 cm^2^/(V·s) cm, respectively, and a quite low resistivity (ρ) = 2.9 × 10^−4^ (Ω·cm) [97].

**Figure 7:**
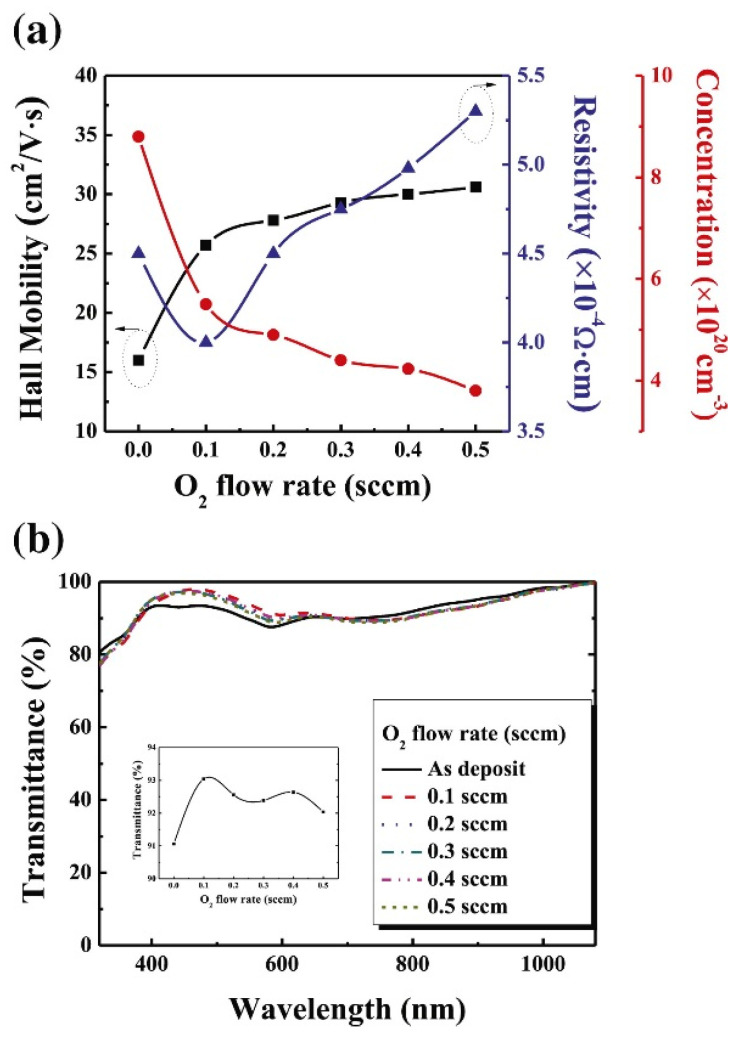
Correlation between (**a**) Hall mobility, resistivity, and carrier concentration and (**b**) optical transmittance of ITO films for different O_2_ flow rates (reprinted with permission from ref. [38]; copyright form Elsevier order number 5492920712562).

**Figure 8:**
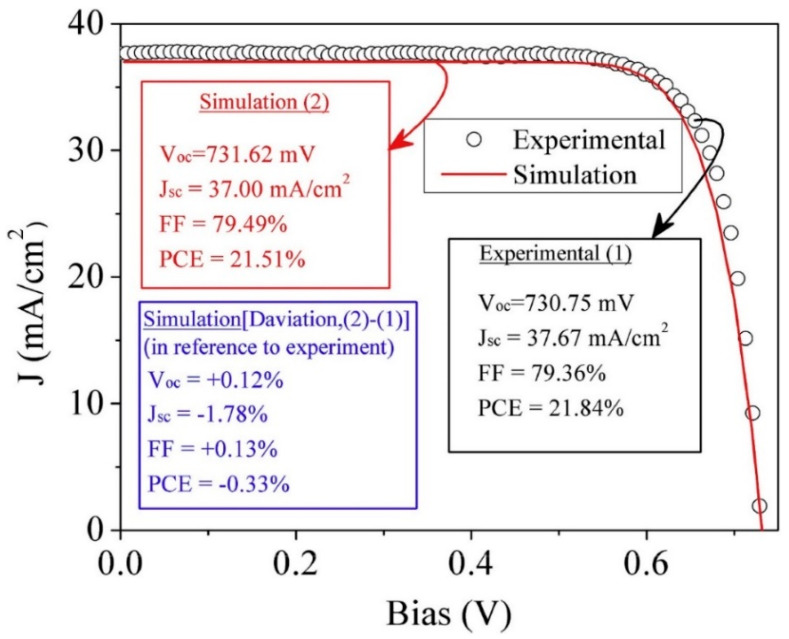
The J-V characteristic plots of a simulated and experimental solar cell (reprinted with permission from ref. [98]; copyright form Elsevier order number 5492920955733).

Figure 7a shows the resistivity, carrier concentration, and mobility of ITO layers for different O_2_ flow rates, and Figure 7b displays the optical transmittance of ITO layers for different O_2_ flow rates [38]. Figure 8 shows the J-V characteristics of an SC, and a small difference is observed in the simulated and experimental SCs. The n-type n-ncSi:H was applied as the FSF in a real SC, where the efficiencies of the simulated SC and the reference cell were 21.51% and 21.84%, respectively. The investigators suggested that if an 80 nm thick ITO were applied as FSF, the PEC of the cell could reach up to 23.8%; however, if a 20 nm thick ITO was used as the FSF, then the cell could achieve PEC of 25.67% [98]. 

Adachi et al. demonstrated that the rear and front TCO contact large area c-Si SHJ SCs showed 25.1% efficiency [99]. Some of the challenges in using doped ITO in SHJ solar cells include achieving high conductivity without sacrificing transparency and ensuring that the doped ITO layer does not degrade the underlying silicon layers. In addition, the cost of the raw materials used to produce doped ITO can be a limiting factor in widespread use. To address these challenges, researchers are exploring new methods for synthesizing and depositing doped ITO films, such as atomic-layer deposition and pulsed-laser deposition. They are also investigating the use of alternative TCO materials, such as zinc oxide and aluminum-doped zinc oxide, which may offer improved performance at lower cost. 

Kim et al. suggested conductive polymers, such as poly(3-hexylthiophene-2,5-diyl): [6,6]-phenyl-C61-butyric acid methyl ester and poly(3,4-ethylene dioxythiophene): poly(styrene sulfonate) to replace ITOs in flexible devices. However, the mechanical properties and electrical efficiency of these polymers are low, compared to ITOs [85]. The introduction of a single metal layer to an ITO film (ITO/metal and metal/ITO) is considered to increase ITO properties. In this context, only a few reports are available on bilayer combinations, so future research should be focused on the fabrication process [89]. 

One additional strategy, the fabrication of sandwich-structure TCO electrodes by RF and DC magnetron sputtering, gained much attention due to its low synthesis cost, high electrical conductivity, transmittance, flexibility, and superior mechanical properties with single-layer transparent electrodes [100]. Thus, TCO electrodes based on sandwich structures with excellent properties, such as silver-nanowire-structure electrodes, have been applied in high-efficiency solar cells [101]. 

Additionally, graphene material has huge potential for the development of TCO materials because of its excellent light transmittance and high electrical conductivity [88]. Table 2 provides a list of TCO films and deposition techniques with the films’ parameters, properties, and efficiency, respectively [31,32,33,34,36,38,53,78,91,93,94,95,97,98]. In terms of outlook, the development of high-performance and low-cost TCO materials is crucial for the commercialization of SHJ solar cells and other high-efficiency solar cell technologies. Doped ITO remains a widely used and well-established material, but continued research is needed to address the challenges that are associated with its use and to explore alternative materials that may offer improved performance at lower cost.

## 5. Conclusions

With the potential for providing inexpensive, ultra-lightweight, and flexible optoelectronic applications such as solar cells, LEDs, and OLEDs, transparent conducting oxides (TCO) are in the limelight. TCO films can be deposited through various deposition methods. The RF magnetron sputtering method is widely applied to fabricate TCO layers, due to the low deposition temperatures, the high efficiency, and the good uniformity of obtained thin films. In addition, layer characteristics can be tuned by changing the sputtering conditions, such as the RF power, the substrate temperature, and the O_2_ flow rate. The doped ITO thin films, such as ITO:Zr, double layer ITO/In_2_O_3_, ZnO:B, Mo, Ti, W, and Nb-doped ITO electrodes, show high transmittance, high conductivity, and mobility suitable for efficient silicon heterojunction (SHJ) solar cell application. The ITO thin film layers can be used as the front and back of the electrode for SHJ solar cells. Hence, the doped In_2_O_3_ materials, such as IO:H, IWO, and ICO:H, are quite promising substitutes for ITO for highly efficient SHJ solar cells.

## Figures and Tables

**Table 1:** The deposition of ITO thin films by various techniques, along with the advantages and disadvantages of each technique.

Materials	Deposition Technique	Advantages	Disadvantages	Refs.
ZnO	Metalorganic chemical vapor deposition (MOCVD)	Increased Hall mobility reduced electron trap density of TCO’s.	Small domain size and carbon contamination, low carrier concentration.	[14]
ZnO:H	Atomic layer deposition (ALD)	Low processing temperature, stoichiometric control, a great degree of crystallinity, higher optical band gap.	Complicated reaction chemistry of TCO, Not good for multicomponent TCO films, poor step coverage, corrosion problems.	[15]
ITO	Thermal evaporation	High deposition rate, high crystallinity, high purity.	Complex geometries.	[36]
ITO	RF and DC Magnetron sputtering	High deposition speed, used for different metals, alloys, and oxides, large-area deposition, reproducibility, high Carrier concentrations, high purity, low sheet resistance of TCO’s.	Very low target utilization, and plasma instability during deposition.	[38]
ITO	Spray pyrolysis	Low-cost fabrication of TCO films, single-step process, non-vacuum, large area applications, produce, alow precursor volume, low temperature, various nanostructures.	Low yield of TCO, difficulties with determining the growth temperature of TCO, Non-uniformity of TCO films.	[40,41]
In_2_O_3_	Pulsed-laser deposition (PLD)	Control over TCO Composition, versatility, and high deposition rate for TCO.	High fabrication cost of TCO, the low carrier mobility of TCO, limited for small substrates, low average deposition rate, and large defects in TCO film.	[42]
ITO	Chemical vapor deposition (CVD)	Doesn’t require a high vacuum, coating of TCO on complicated shapes, low resistivity, the high optical transmittance of TCO’s.	Challenging to synthesize multi-component materials.	[47]

**Table 2:** List of TCO films and deposition techniques with parameters, films properties, and efficiencies.

Materials	Deposition Technique	Parameters		Film Properties	Performance Parameters				Ref.
		Deposition Temp (°C)	Gas Flow Rate	Resistivity (Ω cm)	J_SC_ (mA/cm^2^)	V_OC_ (mV)	Fill Factor (%)	Efficiency (%)	
ITO:Zr	RF-magnetron sputtering	200	1.5 × 10^−3^ Torr	4.39 × 10^−4^	33.66	710	72.4	17.31	[31]
IWO	Plasma enhanced chemical vapor deposition (PECVD)	150	0.4 Pa	2.8 × 10^−4^	38.56	727.8	78.48	22.03	[32]
ICO:H	high-density plasma-enhanced evaporation	150	0.45 Pa	2.21 × 10^−4^	38.8	745	83.2	24.1	[34]
ITO	Reactive thermal evaporation (RTE)	160	-	3.74 × 10^−4^	9.31	1660	68	10.51	[36]
ITO	RF-magnetron sputtering	200	1.5 × 10^−3^ Torr	5.3 × 10^−4^	35.1	665	73.2	17.1	[38]
ITO	RF-magnetron sputtering	250	1 × 10^−6^ Torr	1.85 × 10^−4^	29.00	613	72	12.80	[52]
ITO:Zr	Thermal evaporation	120	-	1.6 × 10^−4^	11.31	875	70.90	7.02	[77]
ITO	RF-magnetron sputtering	200	1.5 × 10^−3^ Torr	3.8 × 10^−4^	34.91	650	71.6	16.3	[90]
ITO/In_2_O_3_	RF-magnetron sputtering	27	2.3 × 10^−3^ Torr	1.72 × 10^−4^	37.42	670	71.16	17.84	[92]
ZnO:Al	Atomic layer deposition (ALD)	80	-	1.4 × 10^−3^	-	750	76.4	21	[93]
ZnO:Al	RF-magnetron sputtering	27	2.7 × 10^−3^ mbar	-	-	735	74.7	19.5	[94]
ZnO:B	Metal organic chemical vapor deposition (MOCVD)	160	50 Pa	7.12 × 10^−3^	31.4	694	76	16.5	[96]
IO:H	RF-magnetron sputtering	27	1 × 10^−4^ Pa	2.3 × 10^−4^	34.26	612	76	16.06	[97]

## Data Availability

Not applicable.
